# Reptarenaviruses in Wild- and Captive-Origin Boid and Pythonid Snakes in Peru

**DOI:** 10.3390/ani16193112

**Published:** 2026-10-03

**Authors:** Jose Antonio Otero Soberon, Steven B. Tillis, Jose Antonio Otero Corbetto, Michael Gulman, Joaquin Vasquez, Robert J. Ossiboff

**Affiliations:** 1 Department of Comparative, Diagnostic, and Population Medicine, College of Veterinary Medicine, University of Florida, Gainesville, FL 32608, USA; 2 Zoocriadero El Huayco, Lima 15457, Peru; 3 Centro de Ornitologia y Biodiversidad (CORBIDI), Lima 15023, Peru; 4 Instituto Peruano de Herpetologia (IPH), Lima 15038, Peru

**Keywords:** *Arenaviridae*, boa, boid inclusion body disease, python, snake, virus

## Abstract

Viral pathogens represent an increasing threat to wildlife health and biodiversity. Most studies of reptile viruses are in captive populations, while knowledge of viral occurrence and diversity in free-ranging reptiles is limited. Some reptarenaviruses (family *Arenaviridae*) are causative agents of boid inclusion body disease (BIBD), an important disease of captive boas and pythons that can be associated with substantial morbidity and mortality in some species. Peru is home to both diverse boid snakes and significant illegal reptile trade. This represents a unique and uninvestigated opportunity for reptarenaviral surveillance to better understand the presence of these viruses in boas in Peru. Oral swabs from 28 individual snakes representing four native boa taxa (*Boa constrictor constrictor*; the endemic subspecies *B. c. ortonii*; *Epicrates cenchria*; and *Eunectes murinus*), one non-native boa taxon (*B. imperator)*, and two non-native python taxa (*Python bivittatus* and *Python regius*) were screened for reptarenaviruses using reverse transcription-PCR and Sanger sequencing. Overall, 64.3% (18/28) of snakes were positive for reptarenaviruses, including 70% (14/20) of wild-origin snakes and 50% (4/8) of captive-origin snakes. Reptarenavirus PCR-positive snakes included both native taxa, including the red-tail boa (*Boa constrictor constrictor*; 64.3%, 9/14 snakes), Orton’s boa (*B. c. ortonii*; 75%, 3/4 snakes), green anaconda (*Eunectes murinus*; 100%, 2/2 snakes), and exotic taxa, including the Central American boa (*B. imperator*; 100%, 2/2 snakes), Burmese python (*Python bivittatus*; 50%, 1/2 snakes), and ball python (*Python regius*; 50%, 1/2 snakes). Of the positive snakes, 14 snakes yielded sequences with 99.6–100% nucleotide identity to reference sequences of *Reptarenavirus giessenae*, one of the best characterized reptarenaviruses documented in association with BIBD. Two additional wild caught red-tail boas yielded reptarenavirus sequences with highest nucleotide identity to unclassified reptarenavirus (S7/S10) strains, and two snakes had mixed chromatograms compatible with amplification of more than one template. These results represent the first molecular confirmation of reptarenaviruses in Peru and suggest that globally significant viruses of captive snakes may be present in wild Peruvian boa populations.

## Simple Summary

Reptarenaviruses (family *Arenaviridae*) are an important cause of disease in captive boas and pythons. Despite their significance in captive snakes, there is limited data on their occurrence in free-ranging snake populations. Peru is home to both extraordinary free-ranging reptile diversity, including boid snakes, and significant illegal reptile trade both into and out of the country. This interface between captive and wild snake populations represents a unique opportunity for reptarenaviral surveillance to better understand the presence of these viruses in boas in Peru. We screened oral swabs collected from seven snake taxa, including four native taxa, one of which, Orton’s boa (*B. c. ortonii*), is endemic to Peru, and three non-native taxa, for the presence of reptarenaviruses by PCR and Sanger sequencing. Reptarenaviruses were detected in swabs from more than half of the snakes tested, including recent wild-caught snakes (some sampled only days after collection). Most detected viruses were genetically identical to corresponding regions of reference genomes of *Reptarenavirus giessenae*, one of the best characterized reptarenaviruses in captive snakes globally. These results represent the first molecular confirmation of reptarenaviruses in Peru and suggest that globally significant viruses of captive snakes may be present in wild Peruvian boa populations.

## 1. Introduction

Arenaviruses are recognized primarily as zoonotic rodent viruses associated with syndromes of hemorrhagic fevers, such as Lassa and Junín fever. However, in the last fifteen years, increased investigation utilizing advanced molecular techniques has led to the discovery of novel and unique viruses in non-mammalian hosts, including reptiles and fish [1]. Currently, there are five genera within the family *Arenaviridae*: *Antennavirus*, *Mammarenavirus*, *Hartmanivirus*, *Reptarenavirus* and *Innmovirus*. *Mammarenaviruses* include pathogens of mammals, and reptarenaviruses include pathogens of reptiles [2,3]. Both hartmaniviruses and reptarenaviruses are detected primarily in snakes, particularly boas and pythons [4,5].

Reptarenaviruses are enveloped viruses with a single-stranded, bisegmented RNA genome. The genome consists of two segments, S and L, that utilize an ambisense coding strategy. The S segment encodes the nucleoprotein in a negative-sense orientation and the glycoprotein precursor in a positive-sense orientation. The L segment encodes the RNA-dependent RNA polymerase in a negative-sense orientation and a zinc finger matrix protein in a positive-sense orientation [1]. Hartmaniviruses are unique in their lack of a coding region for a zinc finger matrix protein found in other arenaviruses [6,7]. Five species of reptarenavirus are recognized by the ICTV: *Reptarenavirus aurei*, *Reptarenavirus californiae*, *Reptarenavirus commune*, *Reptarenavirus giessenae*, *and Reptarenavirus rotterdamense* [8].

Reptarenavirus infection is associated with boid inclusion body disease (BIBD), a potentially fatal condition of captive boas and pythons [3]. As its name suggests, BIBD is histologically characterized by eosinophilic cytoplasmic inclusions in epithelial cells, neurons, and leukocytes. The presence of clinical signs in snakes with reptarenavirus infections is variable, but when present are primarily neurologic in nature and can include torticollis (abnormal twisted neck posture), imbalance, opisthotonos (severe arching of the head, neck, and body), flaccid paralysis, and lack of a righting reflex (inability to right the body when placed in dorsal recumbency) [4,9]. In boids, the disease is often subclinical or very slowly progressive and generally accompanied by a low mortality rate; however, in pythons, the development and progression of neurological signs is rapid and often fatal [10]. Transmission of reptarenavirus is poorly understood, but vertical transmission has been demonstrated in captive snakes [11].

Reptarenaviruses have been detected in captive snake collections (private and zoological) from North America, Europe, Asia, Australia, and, more recently, South America [9,12]. The first documentation of reptarenaviruses in free-ranging snakes was reported in wild, native *Boa constrictor* in Costa Rica from samples collected as early as 1989 [3]. Reptarenaviruses have also been detected in wild caught boid snakes maintained in captivity in Brazil [9]. Together these findings suggest that at least some reptarenaviral lineages may be present in free-ranging boas in the Neotropics in addition to captive snakes globally.

**Figure 1:**
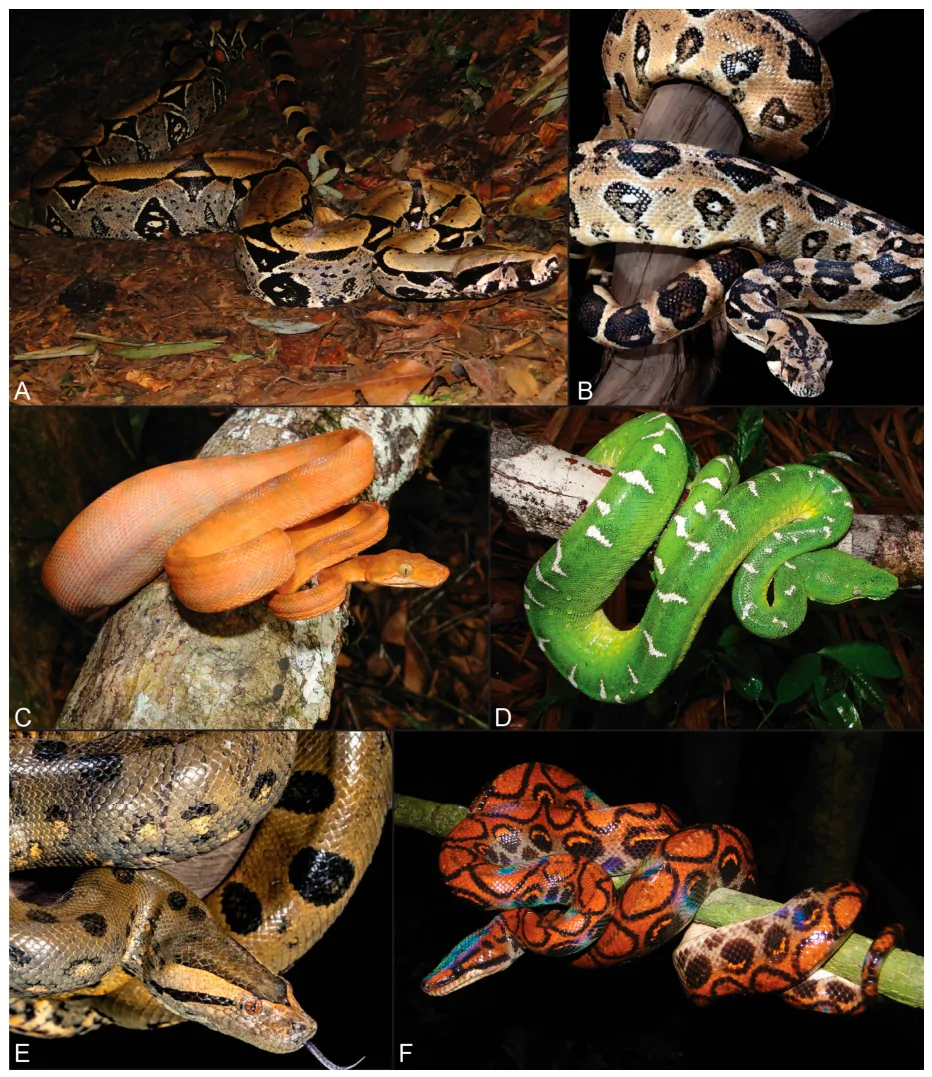
Boid snakes native to Peru. (**A**) Red-tail boa constrictor (*Boa constrictor constrictor*). (**B**) Orton’s boa (*Boa constrictor ortonii*). (**C**) Amazon tree boa (*Corallus hortulanus*). (**D**) Amazon basin emerald tree boa (*Corallus batesii*). (**E**) Green anaconda (*Eunectes murinus*). (**F**) Rainbow boa (*Epicrates cenchria*). Photographs courtesy of Michael Tweedle and Pablo Venegas.

Peru is a country of incredible biodiversity, with more than 25,000 native species of plants and animals [13]. Hundreds of reptile species are native to Peru, including over 440 lizard and snake species, 20 turtle and tortoise species, and five crocodilian species [14]. The diversity of boid snake species in the region is particularly notable, with six taxa (Figure 1), including the red-tail boa constrictor (*Boa constrictor constrictor;* Figure 1A), Orton’s boa (*Boa constrictor ortonii*; Figure 1B), Amazon tree boa (*Corallus hortulanus*; Figure 1C), Amazon basin emerald tree boa (*Corallus batesii;* Figure 1D), green anaconda (*Eunectes murinus*; Figure 1E), and rainbow boa (*Epicrates cenchria*; Figure 1F), all having native ranges within Peru. The utilized taxonomic convention recognizes the species *Boa constrictor* to comprise the subspecies *B. c. constrictor* and *B. c. ortonii*, as distinct from the species *Boa imperator*. With the exception of Orton’s boas (*B. c. ortonii*), these Peruvian boids are also commonly encountered in the pet trade, from both wild caught and captive bred origins.

Given the incredible and unique biodiversity of Peru, it should not be surprising that substantial conservation issues related to illegal internal and international wildlife trafficking also exist [15]. Thousands of animals are captured and transported under suboptimal to dangerous conditions annually. While only approximately 80 snakes were reported to be seized by Peruvian authorities as a result of illegal trade in 2024, this represents only a small fraction of the total number of animals trafficked each year [16]. In addition to having direct negative effects on animal health, such animal movements can also facilitate pathogen movement and translocation within and out of the country [17].

Costa Rica, Brazil, and Peru all possess related native snake species and partially overlapping ecological niches. Given that reptarenaviruses have been detected in free-ranging boas in Costa Rica and wild-caught boids from Brazil, we hypothesized that reptarenaviruses were also likely present in free-ranging boas in Peru. Additionally, anecdotal reports of BIBD-like disease exist in captive boid snake populations in Peru. Given the prominence of wildlife trafficking in Peru, the high number of boid snakes native to the country that are also common in the pet trade from both wild and captive origins, and anecdotal reports of BIBD-like disease, we also hypothesized that reptarenaviruses would also be present in captive snakes in the country. To test these hypotheses, we screened boid and pythonid snakes in Peru representing native, endemic and exotic taxa of three origins (snakes recently collected from the wild; snakes with wild-caught origins but maintained as captive animals long-term; and captive bred snakes) for reptarenaviruses by reverse transcription polymerase chain reaction (RT-PCR) followed by Sanger sequencing.

## 2. Materials and Methods

To assess the presence of reptarenavirus nucleic acids in samples collected from snakes in Peru, orochoanal swabs were collected from both native boas and exotic boas and pythons in December 2024. Samples were collected from both snakes at Zoocriadero El Huayco, a rescue and rehabilitation center in Huachipa, Lima, Peru, and from private collections in other parts of Lima. A total of 28 snakes were sampled, representing three boa species, one anaconda species, and two python species (Table 1). Twenty snakes had wild origins (wild caught), with varying lengths of time between wild collection and sampling, ranging from approximately seven days to over two years. Eight additional snakes were sampled that were born in captivity and maintained in captive collections. Sample collection was made following Peruvian government protocols with the appropriate permits (Peru Servicio Nacional Forestal y de Fauna Silvestre [SERFOR] AUT-IFS-2025-088), animal welfare standards, and utilizing strict biosafety measures. Each snake was carefully removed from its enclosure or holding container with a herpetological hook, and its head was gently restrained manually. To prevent cross-contamination between snakes, the hook was rinsed with water to remove organic debris and then disinfected with a commercial disinfectant (Ox-Virin; Grupo Ox, Huesca, Spain) at a 1% working dilution with a minimum contact time of 5 min, and nitrile gloves worn by all personnel involved in sample collection were changed between snakes. Two sterile surgical hemostatic forceps were used in rotation to assist in exposing the oral cavity, allowing one forceps to complete disinfection while the other was in use; each was rinsed and disinfected between snakes as previously described. Forceps were used only to expose the oral cavity and were kept away from the mucosal areas subsequently sampled by swabbing. A sterile swab was vigorously rubbed across the mucosa of the dorsal oral cavity and choana, base of the oral mucosa, and around the glottis in a single motion, in an effort to minimize snake handling time. Swabs were placed into sterile microcentrifuge tubes, labeled with patient identification, and then transferred to a cooler with abundant dry ice to minimize RNA degradation, and immediately transported to the laboratory for nucleic acid extraction upon arrival.

Sample extraction, PCR analysis, and gel electrophoresis were conducted in the molecular biology laboratory at the Zoocriadero El Huayco, which was established specifically for this investigation due to the limited availability of veterinary molecular diagnostic laboratories in Peru. Total RNA was extracted from each swab using the RNeasy Purification Kit (Qiagen, Germantown, MD, USA) per manufacturer’s instructions; a negative extraction control was also extracted for each batch (*n* = 7) of swabs extracted. Eluted nucleic acids were stored in cryotubes in liquid nitrogen (−196 °C) until RT-PCR was performed. Swab extracts were screened for reptarenaviruses using the One-Step RT-PCR Kit (PCR Biosystems, East Hartford, CT, USA) and previously reported primers targeting a portion of the S-segment glycoprotein gene, MDS-400 (5′-TTCATTTCTTCATGRACTTTRTCAATC-3′) and MDS-402 (5′-GGSATAACAAAYTCACTTCAAATATC-3′) [4]. The final 25 µL reaction volume for each sample contained 12.5 µL of 2× RT-PCR Master Mix, 2 µL each of 10 µM MDS-400 and MDS-402 primer stocks, 1.5 µL reverse transcriptase, 3 µL of RNA extract, and 4 µL nuclease-free water. PCR cycling was performed in a Veriti 96 well thermal cycler (Applied Biosystems, Waltham, MA, USA) following the previously reported protocol by Chang et al., (2016) [4]: 50° C for 15 min; 95 °C for 5 min; 40 cycles of 95 °C for 30 s, 52 °C for 30 s, and 72 °C for 30 s; and 72 °C for 7 min followed by holding at 4 °C. All swab extracts and negative extraction control were screened, and a water-only negative control was also included in each RT-PCR batch to identify potential contamination. No known positive control was available for use in country; however, following Sanger sequencing at least one sequence confirmed-positive sample was present in each sample batch run. No internal/host extraction control was performed.

PCR products were resolved by gel electrophoresis using 1% agarose gels prepared in 1× TBE buffer and run at 100 V for approximately 40 min with a 100 bp DNA ladder (PCR Biosystems, East Hartford, CT, USA). Gels were stained with ethidium bromide and visualized with an ultraviolet transilluminator. Bands of the expected size (~275 bp) were excised, gel purified using the QIAquick gel extraction kit (Qiagen, Germantown, MD, USA) and stored at −20° C for up to 25 days.

Extracted PCR amplification products from bands of the expected size were transported to the USA via commercial airline on ice packs and briefly stored at −20° C prior to submission for bidirectional commercial Sanger sequencing (Azenta Life Sciences, South Plainfield, NJ, USA). Sanger sequences were assembled, primer sequences were removed, and sequences were analyzed using commercial software (Geneious Prime ver. 2024.0.7; Biomatters, Auckland, New Zealand).

## 3. Results

Orochoanal swabs from nearly two-thirds (64.3%; 18/28) of the snakes in this study were RT-PCR positive for reptarenaviruses (Table 1). Of the native, wild caught snakes, 70% (14/20) were reptarenavirus PCR positive. This included red-tail boas (*B. c. constrictor*; 75%, 9/12 snakes), Orton’s boas (*B. c. ortonii*; 75%, 3/4 snakes), and green anacondas (*E. murinus*; 100%, 2/2 snakes). While many of these snakes were maintained in captive conditions for 12 months or more at the time of sampling, two snakes, an Orton’s boa (*B. c. ortonii*) and a red-tail boa (*B. c. constrictor*), were sampled approximately 7 and 10 days post collection from the wild, respectively, and had neither documented direct nor indirect contact with other captive or collected snakes prior to sampling. Two additional red-tail boas that were produced in captivity were negative for reptarenaviruses.

Reptarenaviruses were also detected in 75% (4/6 snakes) of the non-native snakes sampled, including Central American boas (*B. imperator*; 100%, 2/2 snakes), Burmese pythons (*P. bivittatus*; 50%, 1/2), and ball pythons (*P. regius*; 50%, 1/2 snakes). All non-native snakes were born in captivity either in Peru (*n* = 3) or internationally (*n* = 3).

Of the 18 positive swabs, 16 samples contained a single consensus sequence with clear chromatogram peaks 234 bp in length after primer editing. BLAST analysis identified all sequences as the appropriate targeted region of the reptarenavirus S segment glycoprotein. Twelve sequences (67% of RT-PCR positive samples; Snakes 2, 5, 7, 9–11, 15–17, 19, 26–27 [Table 1]), were 100% identical to each other and to reference sequences of *Reptarenavirus giessenae*, including reference GenBank accessions for University of Giessen reptarenaviruses -1 (MH483061), -2 (KR870015), and -3 (KR870013). This positive cohort included both wild-caught native Peruvian boids (*B. c. constrictor*, *n* = 6, snakes 2, 5, 7, 9–11; *B. c. ortonii*, *n* = 2, snakes 17&19) and captive-born exotic boid and pythonid snakes (*B. imperator*, *n* = 2, snakes 15&16; *P. bivittatus*, *n* = 1, snake 26; *P. regius*, *n* = 1, snake 27). Two additional samples, both from wild-caught green anacondas (*E. murinus*; snakes 23&24) were 100% identical to each other, and 99.6% identical to reference sequences of *Reptarenavirus giessenae*. A representative of each unique sequence was deposited in GenBank (PZ844034 and PZ844035).

Two wild-caught red-tail boas (*B. c. constrictor*; snakes 3&4) were positive for a 234 bp reptarenaviral fragment with 100% identity between the two, and 99.2% identical to unclassified S7-like and S10-like reptarenaviruses (MH483088 and MH503957, respectively; GenBank Accession PZ844036). Lastly, in two boas (*B. c. constrictor* [snake 6] and *B. c. ortonii* [snake 20]), sequences generated from appropriately sized amplicons had overlapping chromatograms; the consensus sequence was most closely related to reptarenavirus accessions in GenBank (88–90% nucleotide identity), but mixed amplification of either different reptarenaviruses or a reptarenavirus and non-target nucleic acid prevented further characterization.

## 4. Discussion

This study expands the documentation of reptarenaviruses in wild origin and captive snakes in Central and South America and reinforces the need for disease monitoring of both captive-managed and free-ranging snakes in Peru. Of the seven snake taxa screened, six taxa (85.7%) were positive for reptarenavirus nucleic acids by RT-PCR. This included three taxa native to Peru (red-tail boas, *B. c. constrictor*; Orton’s boas, *B. c. ortonii*, endemic to Peru; and green anacondas, *E. murinus*) and three non-native taxa (Central American boas, *B. imperator*; Burmese pythons, *P. bivittatus*; and ball pythons, *P. regius*). The most common virus sequences detected in our study population (77.8% [14/18] positive samples) exhibited very high nucleotide identity (99.6–100%) to *Reptarenavirus giessenae* reference sequences. This finding is both unexpected and relevant, as *Reptarenavirus giessenae* is arguably the most characterized reptarenavirus and one of the most common lineages detected in captive animals worldwide [18]. This virus was detected in both exotic captive-born snakes as well as native, wild-caught snakes. The identification of *Reptarenavirus giessenae*-like S-segment sequences in CB exotic snake species produced in the pet trade, while the first time documented in Peru, is not surprising. It is similarly unsurprising that such viruses would also be identified in a mixed collection of CB and WC snakes (most maintained in captive conditions for seven months or greater; Snakes 5, 7, 9–11, 19, 23–24). However, the identification of similar viruses in freshly caught wild snakes, a red-tail boa (*B. c. constrictor*, snake 2) and an Orton’s boa (*B.c. ortonii*, snake 17) was not anticipated. These snakes had been collected from the wild only 7–10 days prior to sample collection and brought to Zoocriadero El Huayco by SERFOR. Upon receipt of these snakes at the center, they were placed individually in disinfected plastic tub-like enclosures with paper as a substrate and were maintained under strict biosecurity guidelines to limit potential transmission of pathogens from the wild snakes to other snakes housed at the facility and vice versa. This finding suggests the possibility that *Reptarenavirus giessenae* and/or related viruses circulate in wild boas in Peru.

While additional studies of larger numbers of samples collected from wild Peruvian boids are necessary to confirm this hypothesis, the short period of time between collection and sampling and the strict biosecurity measures enacted are compatible with pre-existing *Reptarenavirus giessenae*-like infections prior to their arrival at the center for sampling. Unfortunately, there are no published studies that document the timeline that reptarenaviruses can be detected from orochoanal swabs in experimentally infected boas. In two studies of experimental reptarenavirus infection in snakes, ball pythons inoculated with different doses of *Reptarenavirus giessenae* were RT-PCR positive in their brain by 14 days post-inoculation (intracoelomic and intratracheal administration) [19], while boa constrictors inoculated with *Reptarenavirus californiae* had detectable viral RNA in numerous tissues by the first testing point 35 days post-infection (intracardiac inoculation) [10]. Given the lack of reptarenavirus infection timeline data on time of detection of reptarenavirus from orochoanal swabs post-infection, post-collection *Reptarenavirus giessenae*-like exposure cannot be entirely ruled out.

**Figure 2:**
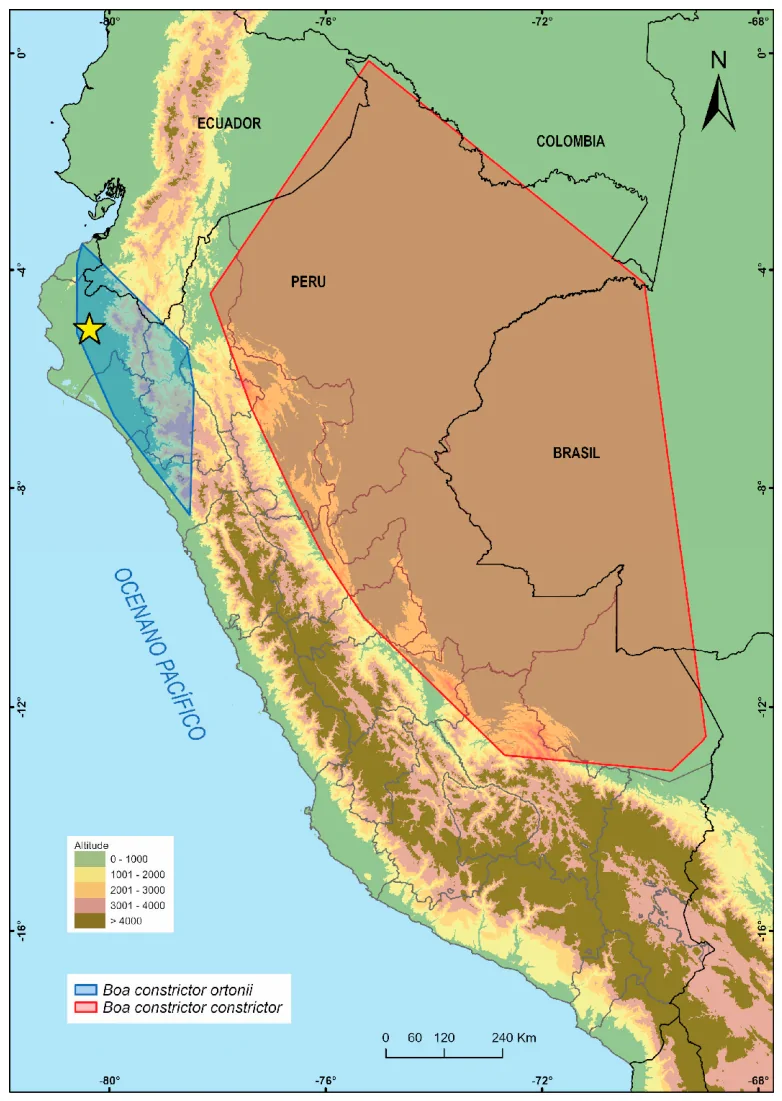
The native ranges of Orton’s boas (*Boa constrictor ortonii*) and red-tail boas (*Boa constrictor constrictor*) in South America. Range polygons were derived from the herpetological collection database of the Instituto Peruano de Herpetología (IPH). The ranges of Orton’s boas (shown in blue) and red-tail boas (shown in red) are distinct and non-overlapping. The yellow star highlights the region where the recently collected Orton’s boa RT-PCR positive for *Reptarenavirus giessenae*-like S-segment sequence originated. Map courtesy of Luis Achay.

If both recently captured, *Reptarenavirus giessenae*-like PCR positive wild boas represent true free-ranging infections, then one of the next logical questions is whether such infections represent circulation of reptarenavirus in wild Peruvian boids or spillover of these viruses from captive snakes in Peru into the wild. The detection of *Reptarenavirus giessenae*-like S-segment sequences specifically in a recently collected Orton’s boa (*B. c. ortonii*) suggests that, at least in some parts of Peru, these viruses may circulate in free-ranging boas. Orton’s boas are a subspecies of Boa constrictor native to the northern coast of Peru [20]. The range of this boa subspecies is distinct from its relative, the red-tail boa (*B. c. constrictor*), which inhabits the Amazon and has a much wider distribution in multiple countries (Figure 2). In contrast, the range of Orton’s boas is geographically isolated in environs that are difficult to access; this likely contributes to the rarity of these snakes in captivity globally. Unlike red-tail boas, which are common in the pet trade and are a component of the legal and illegal reptile trade in Peru, Orton’s boas are to the best of our knowledge rarely encountered in the exotic pet trade. This rural region of Peru is also not known to experience a high influx of exotic reptiles. Together, these characteristics make an exotic introduction of reptarenaviruses or reptarenavirus-infected snakes less likely and adds additional weight to the argument that *Reptarenavirus giessenae* may circulate in free-ranging boas in Peru; however, additional sampling of boas in the wild is necessary to confirm this hypothesis.

The hypothesis that *Reptarenavirus giessenae* is present in wild boas in Peru also gains some support by the identification of the virus in orochoanal swabs collected from two wild-caught green anacondas (*Eunectes murinus*; snakes 23&24). While both of these snakes were maintained in captivity for 7 months or longer, the reptarenavirus sequence detected in their orochoanal swabs was 100% identical to each other but contained a unique single nucleotide polymorphism not represented in other snakes at the facility or in over 200 GenBank accessions of *Reptarenavirus giessenae*. This sequence variant may represent previously uncharacterized reptarenaviral diversity, although its origin cannot be determined. The possibility that *Reptarenavirus giessenae* is circulating in wild populations of boids in Peru bears more significance considering that Peru is recognized as one of the main exporters of reptiles worldwide via both legal and illegal trade [21,22]. As such, the movement of snakes from Peru to other parts of the globe may facilitate continued spread of this virus in the pet trade. There is precedent for translocation of reptile pathogens from Peru to Europe via animal movements, as has recently been documented in caiman lizards [17]. However, this finding should be interpreted cautiously given the small number of wild individuals analyzed. Additional sampling of Peruvian boids, particularly Orton’s boas and green anacondas in the field, is necessary to confirm the preliminary findings and hypotheses proposed in this study.

Four exotic snakes of three species were also positive for *Reptarenavirus giessenae*-like S-segment sequences. One snake, a ball python (snake 27) was produced in Peru, while the other three snakes, two Central American boas (snakes 15&16) and a Burmese python (snake 26), were imported into the country from the United States of America and another country in South America, respectively. As legal importation of these exotic species into Peru is subject to stringent regulations [Ley N° 29763; D.S. N° 019-2015-MINAGRI], and the importation of ball pythons (*P. regius*) is specifically restricted, as it is considered an invasive species by the Peruvian government [D.S. N° 006-2022-MINAM], all of these animals originally entered the country illegally. As all smuggled snakes were present at the center for 6 months or more, and many other snakes at the center were also reptarenavirus positive, it is unclear if the snakes were imported with the virus or they acquired it after arrival. However, even the possibility that the illegal trade and trafficking of reptiles into Peru has facilitated the introduction of viruses not previously documented in the country should be considered an epidemiological risk factor [23,24].

Other reptarenaviruses were also detected in the snakes in this study. Two wild-caught red-tail boas were positive for unclassified reptarenaviruses most similar to S7- and S10-like reptarenaviruses characterized from captive boa constrictors in Europe [6]. These viruses were not found in other snakes at the Zoocriadero El Huayco and may also represent wild viral diversity, but as discussed previously, infection after collection cannot be excluded. Two additional snakes, a red-tail boa (snake 6) and an Orton’s boa (snake 20) were positive for reptarenavirus, but overlapping chromatograms limited interpretation of the virus(es) present. While the result does not clearly indicate viral co-infection, reptarenavirus coinfections are recognized in captive snakes with BIBD [25].

Only one of the reptarenavirus positive snakes at Zoocriadero El Huayco, a Central American Boa (*B. imperator*) had a history or active neurological signs at the time of sampling, exhibiting abnormal twisting of the neck and head positioning. However, such clinical signs are common among trafficked snakes seized by Peruvian authorities (Otero, personal observation). Targeted reptarenaviral molecular surveillance in seized and trafficked snakes in Peru to better understand viral prevalence is warranted.

The work presented herein is not without limitations. First, the technical capacities in Peru limited viral analysis to single-locus, conventional RT-PCR only amplifying a small portion of one segment of the reptarenaviral genome. Due to the complexities of CITES permitting and to address regional limitations, a lab was constructed in Peru where initial PCR was performed. PCR amplicons, which are able to be moved internationally without such permits, were then transported to the United States for subsequent sequencing and analysis. This approach can be extremely valuable for wildlife disease surveillance [26], but it is restrictive in that subsequent analyses can be limited. However, such studies can provide critical preliminary data to guide larger scale studies and justify navigation of the oft circuitous process of acquiring CITES permits. To fully interpret and analyze the detected reptarenaviruses, third generation sequencing modalities, including non-biased sequencing, would be of significant value. Unfortunately, access to such technology in Peru, particularly for wildlife studies, is limited. In the future, we hope to augment this study with more in-depth data made possible by legal movement of sample extracts rather than amplified PCR products.

The second limitation of this study is the design of the initial study and the limited history available for the sampled snakes. As even the snakes with the most recent collection history were present in captivity for 7–10 days and were at a facility with documented reptarenaviral infections in the resident snake population, potential exposure to reptarenavirus post-collection cannot be excluded. Future studies hope to assess samples collected in the field to better confirm the presence of reptarenaviruses in free-ranging Peruvian snakes more broadly within the country. Additionally, the original geographic origin of the wild caught specimens included in this study could not be determined, as precise locality data were unavailable. Limited geographic information was obtained for Snake 17 (Figure 2), the wild Orton’s boa positive for *Reptarenavirus giessenae*-like S-segment sequence. However, this information was based solely on informal, undocumented personal communication and lacked fine spatial resolution, only confidently identifying the Peruvian department of origin and not the precise geographic coordinates. No locality data were available for other wild caught snakes, including green anacondas and red-tail boas, as the majority of individuals were confiscated from illegal wildlife trafficking operations, making it nearly impossible to determine their true geographic origin. A final limitation of the study was the lack of an internal host/extraction control, which may have contributed to false-negative results for snakes without a detected reptarenavirus.

The findings reported herein represent the third documentation of reptarenaviruses in snakes of wild origin in Central and South America. Boa constrictors in the neighboring country of Brazil have been documented to have reptarenavirus infections, and the authors suggest a wild origin of those viruses [9]. Samples collected from wild, native *Boa constrictor* in Costa Rica as early as 1989 were documented to be reptarenavirus positive in another study [3]. Together, the findings of this study and previous reports support circulation of reptarenaviruses in free-ranging boids in the Americas. Given the role that Peru and other countries in the Americas play in the export of reptiles around the world [27], international movements of South and Central American reptiles via animal trade may represent one origin of the global dissemination of reptarenaviruses, a possibility that broader phylogeographic sampling could help test. Lastly, this study highlights the potential power of smaller molecular laboratories, like the one established at Zoocriadero El Huayco, in improving understanding of wildlife disease globally, particularly in regions where there is a lack of disease surveillance with concurrent limited access to laboratories equipped to perform such disease testing.

## 5. Conclusions

This study provides the first molecular evidence of reptarenaviruses, predominantly *Reptarenavirus giessenae*-like, in wild-caught and captive boid and pythonid snakes in Peru. Detection of reptarenaviruses in wild-caught Peruvian boids, including recently captured individuals from geographically isolated populations, suggests possible circulation of reptarenaviruses in free-ranging Peruvian boas. Given Peru’s prominent role in the legal and illegal reptile trade, these findings highlight a previously unrecognized risk for the translocation of reptarenaviruses both nationally and internationally. Direct field-based surveillance and complete genomic characterization are warranted to determine the ecological significance of these viruses in free-ranging South American boid populations.

## Figures and Tables

**Table 1:** Native and exotic boid and pythonid snakes screened for reptarenaviruses in Peru.

Snake	Common Name	Scientific Name	Origin	Peru Native?	Captivity Length	PCR and Sequence Result
1	Red-tail boa	*Boa constrictor constrictor*	WC	Yes	~7 days	Negative
2	Red-tail boa	*Boa constrictor constrictor*	WC	Yes	~10 days	*Reptarenavirus giessenae*, 100% nucleotide identity
3	Red-tail boa	*Boa constrictor constrictor*	WC	Yes	>6 months	Unclassified reptarenavirus (S7/S10-like), 99.2% nucleotide identity
4	Red-tail boa	*Boa constrictor constrictor*	WC	Yes	LTC	Unclassified reptarenavirus (S7/S10-like), 99.2% nucleotide identity
5	Red-tail boa	*Boa constrictor constrictor*	WC	Yes	LTC	*Reptarenavirus giessenae*, *100% nucleotide identity*
6	Red-tail boa	*Boa constrictor constrictor*	WC	Yes	LTC	Mixed sequences, consensus reptarenavirus
7	Red-tail boa	*Boa constrictor constrictor*	WC	Yes	LTC	*Reptarenavirus giessenae*, 100% nucleotide identity
8	Red-tail boa	*Boa constrictor constrictor*	WC	Yes	LTC	Negative
9	Red-tail boa	*Boa constrictor constrictor*	WC	Yes	LTC	*Reptarenavirus giessenae*, 100% nucleotide identity
10	Red-tail boa	*Boa constrictor constrictor*	WC	Yes	LTC	*Reptarenavirus giessenae*, 100% nucleotide identity
11	Red-tail boa	*Boa constrictor constrictor*	WC	Yes	LTC	*Reptarenavirus giessenae*, 100% nucleotide identity
12	Red-tail boa	*Boa constrictor constrictor*	WC	Yes	LTC	Negative
13	Red-tail boa	*Boa constrictor constrictor*	CB	Yes	LTC	Negative
14	Red-tail boa	*Boa constrictor constrictor*	CB	Yes	LTC	Negative
15	Central American boa	*Boa imperator*	CB	No	>6 months	*Reptarenavirus giessenae*, 100% nucleotide identity
16	Central American boa	*Boa imperator*	CB	No	>6 months	*Reptarenavirus giessenae*, 100% nucleotide identity
17	Orton’s boa	*Boa constrictor ortonii*	WC	Yes	~7 days	*Reptarenavirus giessenae*, 100% nucleotide identity
18	Orton’s boa	*Boa constrictor ortonii*	WC	Yes	~10 days	Negative
19	Orton’s boa	*Boa constrictor ortonii*	WC	Yes	LTC	*Reptarenavirus giessenae*, 100% nucleotide identity
20	Orton’s boa	*Boa constrictor ortonii*	WC	Yes	LTC	Mixed sequences, consensus reptarenavirus
21	Rainbow boa	*Epicrates cenchria*	WC	Yes	LTC	Negative
22	Rainbow boa	*Epicrates cenchria*	WC	Yes	LTC	Negative
23	Green anaconda	*Eunectes murinus*	WC	Yes	7 months	*Reptarenavirus giessenae*, 99.6% nucleotide identity
24	Green anaconda	*Eunectes murinus*	WC	Yes	LTC	*Reptarenavirus giessenae*, 99.6% nucleotide identity
25	Burmese python	*Python bivittatus*	CB	No	LTC	Negative
26	Burmese python	*Python bivittatus*	CB	No	LTC	*Reptarenavirus giessenae*, 100% nucleotide identity
27	Ball python	*Python regius*	CB	No	>6 months	*Reptarenavirus giessenae*, 100% nucleotide identity
28	Ball python	*Python regius*	CB	No	LTC	Negative

Abbreviations: WC (wild-caught); CB (captive-born); LTC (long-term captive; ≥1 year).

## Data Availability

Unique sequences identified in this study were uploaded to the National Center for Biotechnology Information’s GenBank database (PZ844034-6; https://www.ncbi.nlm.nih.gov/genbank/ (accessed on 10 August 2026)).

## Abbreviations

The following abbreviations are used in this manuscript:

WC	Wild-caught
CB	Captive-born
LTC	Long-term captive; ≥1 year
RT-PCR	Reverse transcription polymerase chain reaction
BIBD	Boid inclusion body disease
SERFOR	Servicio Nacional Forestal y de Fauna Silves
